# Injuries in Aleppo, Syria; first population-based estimates and characterization of predominant types

**DOI:** 10.1186/1471-2458-6-63

**Published:** 2006-03-13

**Authors:** Wasim Maziak, Kenneth D Ward, Samer Rastam

**Affiliations:** 1 Syrian Center for Tobacco Studies, Aleppo, Syria; 2 Department of Health & Sport Sciences, and Center for Community Health, University of Memphis, Memphis, TN, USA; 3 Institute of Epidemiology and Social Medicine, University of Muenster, Muenster, Germany

## Abstract

**Background:**

Despite the growing burden of injuries worldwide, Syria and many other Arab countries still lack population-based estimates of different types of injuries. This study aims toprovide first population-based estimates of major injuries in Syria and characterize groups at increased risk.

**Methods:**

An interviewer-administered population-based survey of adults 18–65 years residing in Aleppo, Syria was conducted in 2004. The study sample involved 2038 household representatives in Aleppo (45.2% men, mean age 35.3 ± 12.1, response rate 86%). We inquired about participants self-reported injuries in the past year that required medical attention as well as injuries among their household members. When reported, injuries were further assessed according to type, place, and outcome.

**Results:**

Overall, there was 153 self-reported injuries in the past year (77.3 per 1000 adult respondents, 93.1 per 1000 in men and 64.4 per 1000 in women, *p = 0.02*). Other than gender, injuries differed by age (the older age group being least affected), and place of occurrence, as men were more likely to sustain traffic injuries and be injured outside the home. Injuries were reported among 236 household members (21.0 per 1000), and were slightly more frequent in children than adults (22.0 per 1000 for children, and 19.7 per 1000 for adults, *p = 0.2*). Traffic injuries, falls, and poisoning (food) were by far the most common types of injury experienced by participants as well as their household members. Falls and traffic injuries seem to have caused most morbidity for the injured, while burns, although not frequently reported, were associated with an unfavorable outcome in the majority of cases.

**Conclusion:**

This information provides baseline information about the burden of different injuries in Syria, and the sociodemographic factors related to them.

## Background

Unintentional injuries represent a major public health risk worldwide accounting for more than 5 million deaths annually and are projected to increase to 8.4 million in 2020 [1,2]. Unintentional injury is the leading cause of death of young people worldwide, and is responsible for 15% of disability adjusted life years (DALYs) lost, making it the third ranking major cause of DALYs lost globally [3,4]. Traffic accidents, a leading cause of injury, are responsible for 1.2 million deaths and 20–50 million injuries/disabilities annually worldwide [5].

The bad news for developing countries is that they bear most of the burden of injuries worldwide. Low and middle income countries account for about 85% of the deaths and 90% of the DALYs lost annually as a result of traffic accidents [5]. The economic cost of injuries is as drastic as the health cost, and is especially burdensome on the already strained economies of developing countries. Estimates suggest that road traffic injuries, in particular, cost low and middle income countries 1% to 1.5% of their gross national product annually. Further, these estimates do not include the hidden costs for affected families and neighborhoods [6,7].

Despite the magnitude of this problem, a population-based surveillance of injuries is still lacking in Syria, as well as most Arab countries. A report on dental injuries among 1087 school children aged 9–12 years in Damascus found that 11.7% of children aged 12 years had some sort of traumatic injuries to their permanent incisors. The two most common causes of these injuries were violence and traffic accidents [8]. The current report aims to address the knowledge gap in this major health problem, utilizing data collected during the Aleppo Household Survey (AHS) conducted in the spring-summer of 2004 by the Syrian Center For Tobacco Studies (SCTS).

## Methods

AHS is a population-based cross sectional survey of a representative sample of adults, 18–65 years of age, residing in Aleppo (total population around 2,500,000 inhabitants). The sample involved 2038 household representatives in Aleppo (45.2% men, mean age 35.3 ± 12.1, age range 18–65 years, response rate 86%), who were able to understand the study procedures, agreed to participate, and provided reliable responses. The slight female predominance in the sample is related to their more availability in the house at the time of survey, although we tried to minimize this factor by making a second appointment when the selected subject was not available in the first run. However, the age distribution of our sample was similar to that of the adult (18–65 years) general population according to the 2004 census [9]. Detailed description of the sampling design and procedures of AHS is reported elsewhere [10,11]. Briefly, relying on the municipality enumeration of residential neighborhoods and number of residents in each neighborhood, a two-stage, stratified, cluster sampling was used, with the target population divided into two strata, formal and informal zones (where residential areas are built illegally on land not zoned for housing). This design was mandated by the fact that about half of Aleppo's area and population live in informal zones, which are likely to have distinctive environmental and health profiles. Within each stratum, residential neighborhoods were randomly selected with probability proportional to size (PPS). Within each neighborhood households were selected with equal probabilities, and within each household one adult in the target age group was randomly selected.

This interviewer-administered survey utilized a computer-based interface for questionnaire and measurement recording. Embedded features were applied to prevent missing or wrong entries, time the procedures, and simplify survey administration. The survey instrument was developed from existing surveys and modified to suit the studied population based on extensive formative work and pilot testing [10,11]. Six, two-person, mixed-gender teams of surveyors participated in one week of training at SCTS on the survey questionnaire, computer interface, data collection procedures, and consent procedures.

Injuries were assessed by asking respondents about injuries (any) requiring medical attention they sustained in the past year. Those responding positively to this question further were asked about the place of injury (home, outside), and type of injury (multiple choice with 9 categories; traffic, fall, fire arm, burn, sharp tool, drowning, poisoning, electric shock, and other). Since physicians in Aleppo use the term "food poisoning" to describe acute gastroenteritis induced by eating contaminated food, the category poisoning refers mainly to this type of injury as was clarified during the formative work preceding the survey [10,11]. We also inquired about the outcome of reported injuries and categorized responses into three outcomes: full recovery (no subsequent consequence), temporary disability (injury related decrease in normal range of activity lasting up to 30 days), and prolonged disability (continuing injury-related decrease in normal range of activity). Respondents also were asked about past year injuries among other household members. When such injuries were reported, the age, gender, place, type, and outcome of injury (with an additional category of death) were recorded (Table 2).

Informed consent was obtained from all study participants.

### Analyses

The final sample was weighted to account for different neighborhood status (formal/informal), multiple neighborhoods, and different number of adults living in households. Sampling weights were calculated according to the method described in [12,13]. All presented figures were calculated from the weighted sample using SPSS 13.0 (complex samples module) [14].

Descriptive statistics were calculated for main study outcomes stratified by gender and age. Information on education, employment, household income, item ownership, and household density (number of individuals living in the household divided by the number of rooms) were combined in a score for socioeconomic status (SES score, range 0–12, with higher values indicating better SES) [11]. Because of small number of some types of injuries, injuries' prevalence was calculated per 1000 (participants or household members). Prevalence of injuries among respondents and their household members were calculated according to the weighted sample as described before. In line with other reports from AHS, age was stratified into three groups and SES score into three tertiles for the purpose of analysis (Tables 1, 2). Chi-square tests were used to assess bivariate relations between injuries and socio-demographic variables for men and women separately (Table 1) and for children and adults separately (Table 2). A logistic regression analyses was used to assess predictors of overall and important subtypes of injuries among participants. Age, gender, education, and SES score were the main covariates entered in the model as shown in (Table 3).

## Results

Overall, there were 153 self reported injuries in the past year (77.3 per 1000) among adult respondents (93.1‰ for men and 64.4‰ for women, *p = 0.02*) reported past year injuries. Injuries differed as well by age (the older age group being least affected), but the difference in injuries according to education, and SES did not reach statistical significance (Table 1). Men were most likely to be injured outside the house while the opposite was true for women (Table 1). Among men, traffic injuries were most common (30.6‰), followed by poisoning (food) (28.2‰), and falls (24.2‰), whereas in women poisoning (food) was most prevalent (24.2‰), followed by falls (20.2‰), and then traffic injuries (19.2‰). Although the sample size precludes arriving at a conclusive assessment of injuries according to their outcome, it appears that most injuries were followed by complete recovery, while falls seem to be most associated with post-injury morbidity.

**Figure 1:**
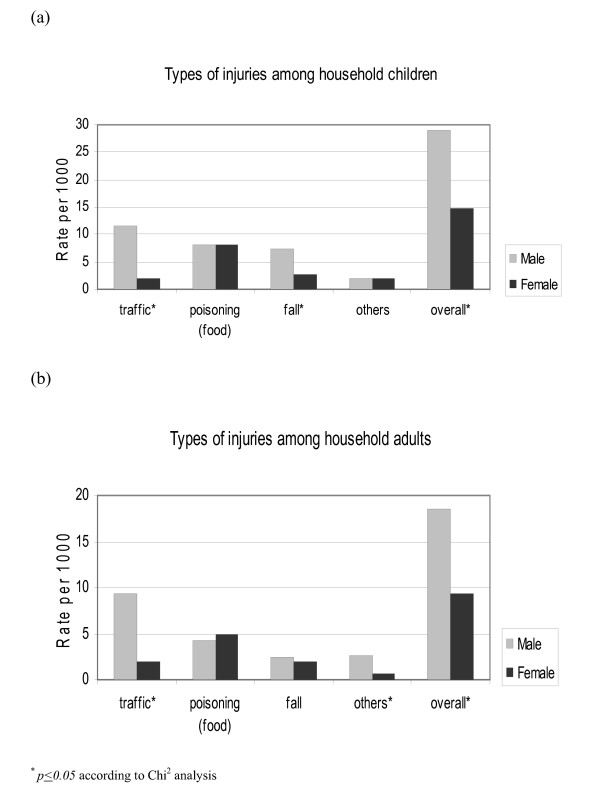
legend: Proportion of different types of injuries and overall injuries by gender among household members (a. children) and (b. adults).

Overall, there was 236 injuries among household members reported (21.0 per 1000) and were more prevalent in children (22.0‰) than adults (19.7‰), but the difference was not statistically significant (*p = 0.2*). Adults were more likely to experience injuries outside of the home, and among children and adults, males were more likely than females to experience injuries (Table 2). Traffic and poisoning (food) injuries were more noted among male household members (children and adults) and topped the list for household members' injuries together with poisoning (food), followed by falls (Table 2, Figure 1). Women household members seem to be proportionately more affected by poisoning (food) accidents than men. Falls again seem to have caused most morbidity for the injured household members (Table 2).

A logistic regression analysis of predictors of injuries among participants showed that gender and age were among the most important, with men being more likely to be injured and to have a traffic injury in particular compared to women, although the difference was significant only at the *p < 0.1 *for all (Table 3). Older participants (46–65 years) on the other hand, were less likely to report injuries in general compared to younger age groups (Table 3).

## Discussion

This is the first report about the spread, types, and outcome of injuries among the general population in Syria. It shows that injuries constitute an important source of morbidity among the population studied, whereby about 8% of adults have reported an injury that required medical attention in the previous year. Among adults, men were more likely to sustain injuries in general, as well as traffic injuries, and to be injured outside the home compared to women. Age also was related to injuries with the oldest age group being least affected. The same gender related trend was seen among household members, where injuries in males were about twice as those in females and where male household member were more likely to be affected by traffic injuries. Respondents and household members who suffered injuries most often reported experiencing a full recovery.

Population-based data about injuries in other Arab countries are scanty, but comparisons can still be made with other developing countries. In a major urban center (Dar es Salam) in Tanzania for example, a population based survey showed that similar to our results men had higher rates of injuries than women and that traffic and falls were major injury types [15]. In Ghana on the other hand, a population based survey showed that the total rate of injuries among adults was similar to our results (76.2 per 1000), with the same gender and type profile in urban Ghanaians as in our population [16]. Because of urban-rural difference in injury rates and profile that are confirmed in other studies [17], our data from Aleppo clearly represent injuries rates and profile of urban adults in Syria.

The strength of this study arises from the fact that it is an interviewer-conducted population-based survey, involving a carefully selected sample that represents adults living in Aleppo. In addition, the use of a computer-based data entry program to record questionnaire responses allowed for error-checking and quality control features which improved the quality of data set. There were limitations, as well. First, information about injuries was obtained by self-report or proxy report (for household members), which allows for recall problems. Poor recall accuracy may underlie the significantly lower prevalence of injuries but higher levels of morbidity reported for household members compared to participants themselves. Participants may have less of an ability to recognize and recall injuries affecting others in the house unless they are of considerable severity, especially given the large household size of the studied communities. So the household prevalence data should be interpreted with caution, as they are likely to be conservative estimates of the real burden of injuries. However, these data are important in asserting the leading types of injuries among the Syrian population. The definition of injuries and their outcome also was based solely on participants' report without validation by medical records, because of the lack of such in Syria. The further the injury in the previous year from the time of survey and the larger the number of injuries the more potential for recall and telescopic biases [18,19]. In our survey more than 90% of those reporting injuries do report one such event (analysis not shown). In addition, poor recall because of length of recall period is expected to be non-differential and affect the rates generally leading to conservative estimates of injuries rates. To improve reporting in general, we applied thorough formative work prior to the survey to adjust the questions and definitions used to the target population [10,11]. For example, a startling finding of this survey was the widespread report of poisoning injuries, which ranked first in prevalence among respondents and second among their household members. However, since we inquired in the survey about injuries require medical attention and that physicians in Aleppo use the term "food poisoning" to describe acute gastroenteritis induced by eating contaminated food, we believe that most of such reported events belong to this type, but acknowledge that we have no mean to support this assumption. This also was supported by the fact that full recovery was the rule for this type of injury. Related to this issue is the season when the survey was conducted (spring, summer), which could have affected this type of events in particular. Warm weather and poor food sanitation and storage are likely to be important contributing factors. Finally, the age frame selected (18–65 years) prevented us from characterizing injuries among the elderly, a particularly important group in the study of injuries.

As noticed in other parts of the world, traffic injuries were among the most common injuries seen in the population studied, mainly affecting men [20]. Men in traditional Arab societies are more mobile compared to women, which also can explain why men received most injuries outside the home compared to women. It is known that restriction on outdoor mobility is more relevant to women compared to children, where mature women either are restricted by societal traditions or by pressing family circumstances (having to look after large households). In fact in this survey, we inquired about average length of time spent indoors and found that women were by large more likely to spend longer times at home compared to men [9].

Falls were also an important type of injury affecting this group, whereby they accounted for approximately one third of the injuries reported by women and one quarter by men. Among household members, falls were reported more often for children than adults. In a hospital-based study of children aged up to 14 years in United Arab Emirates (UAE), injuries were seen more among boys, most often due to falls for children up to five years and traffic accidents for children 5–14 years [21]. In another study among Arab children in Israel, falls were the second most common injury reported at home (after burns) accounting for 28.5% of such injuries [22]. In comparison, burns were reported in frequently in this study (3.2% of household children, analysis not shown). This may be due to the fact that, unlike our urban sample, Broides study involved mobile Bedouin communities likely to be using unsafe methods for cooking and heating [22].

Morbidity and mortality (injuries with fatal outcome among household members) figures due to injury in this study should be interpreted with caution since self reports were not validated by medical records and since the sample size was inadequate for outcome assessment. However, our data point at potential important causes of morbidity due to injury in our population. In this regard, falls and traffic injuries seem to be the cause of most morbidity outcomes in our population. In a hospital-based study of facial injuries in UAE, men were more likely to be injured, while traffic accidents were the most common cause (59%) followed by falls (21.5%) [23]. In another similar study from Sharjah, men were by far more likely to sustain maxillofacial fractures, and motor vehicle crashes were by far the most common cause of these injuries (75%) followed by falls (12%) [24].

The main predictors of injuries in the multivariate logistic regression analysis were age and gender. Men were more prone to injuries in general and to traffic injuries compared to women. This is most likely due to men's higher mobility, longer time spent outdoors, and more use of transportation. Further research looking specifically at each type of injury in its socio-environmental context is clearly warranted to draw a clearer picture of important injury susceptibility factors, especially modifiable ones.

## Conclusion

This study provides the first estimates of injuries among the general population in Syria. It shows that injuries are common among adults in Syria as well as their household members. Main types of injuries identified were traffic, falls, and food-poisoning. Because of greater mobility and more time spent outdoors, men and boys were more prone to injuries, especially traffic. This information provides a baseline orientation about the burden of injuries within the Syrian society and socio-cultural factors likely to be contribute to the injury profile observed. It is hoped that our findings can stimulate and guide future research and intervention work focusing on this major public health problem.

## List of abbreviations

• DALYs- Disability adjusted life years

• AHS- Aleppo household survey

• PPS- Probability proportional to size

• SCTS- Syrian Center for Tobacco Studies

• SES- Socio-economic status

• UAE- United Arab Emirates

## Competing interests

The author(s) declare that they have no competing interests.

## Authors' contributions

W Maziak designed the study and wrote the manuscript, S Rastam did the analyses, KD ward participated in the study's design and revised the manuscript.

## Pre-publication history

The pre-publication history for this paper can be accessed here:



## Figures and Tables

**Table 1:** Prevalence and characteristics of past year self-reported injuries among respondents (18–65 years) in Aleppo-Syria (n = 2038)

	**Men (n = 921)**	**Women (n = 1117)**	**Overall**
Total number of past year injuries and rate (per 1000)*	82 (93.1)	71 (64.4)**	153 (77.3)
Age			
18–29 years	31 (101)	28 (65)	59 (80)
30–45 years	40 (118)	33 (73)	73 (93)
46–65 years	11 (53)^†^	10 (47)	21 (50)^†^

Education			
Illiterate	9 (55)	13 (32)	22 (39)
≤ 9 years	52 (103)	43 (77)	95 (90)
> 9 years	21 (90)	15 (73)	36 (81)

SES			
Lower tertile	18 (102)	37 (53)	55 (63)
Middle tertile	38 (101)	22 (77)	60 (89)
Higher tertile	26 (83)	12 (70)	38 (78)

***Place of injury (non-weighted % of all injuries)***			

Home	22 (26.8)	37 (52.1)	59 (38.6)
Outside	60 (73.2)^†^	34 (47.9)	94 (61.4)^†^

Type of injury(number and rate per 1000)*			

Traffic			
Outcome (non-weighted % of total)	27 (30.6)	18 (19.2)**	45 (24.3)
Recovery	24 (88.9)	18 (100)	42 (93.3)
Disability (temporary)	2 (7.4)	0 (0)	2 (4.4)
Disability (prolonged)	1 (3.7)	0 (0)	1 (2.2)

Fall			
Outcome (non-weighted % of total)	20 (24.2)	22 (20.2)	42 (22.0)
Recovery	15 (75)	18 (81.8)	33 (78.6)
Disability (temporary)	3 (15)	3 (13.6)	6 (14.3)
Disability (prolonged)	2 (10)	1 (4.5)	3 (7.1)

Poisoning (food)			
Outcome (non-weighted % of total)	26 (28.2)	30 (24.2)	56 (26.0)
Recovery	26 (100)	30 (100)	56 (100)
Disability (temporary)	0 (0)	0 (0)	0 (0)
Disability (prolonged)	0 (0)	0 (0)	0 (0)

Fire arm, sharp tool, burn, electric and others			
Outcome (non-weighted % of total)	9 (10.2)	1 (0.7)**	10 (5.0)
Recovery	6 (66.7)	0 (0)	6 (60)
Disability (temporary)	1 (11.1)	0 (0)	1 (10)
Disability (prolonged)	2 (22.2)	1 (100)	3 (30)

*All prevalence estimates are calculated from the weighted sample as described in the analysis section. **p ≤ 0.05 according to Ch^2 ^analysis for the relation between injuries and gender (rows) ^†^*p ≤ 0.05 *according to Ch^2 ^analysis for the relation between injuries and socio-demographic variables (columns)

**Table 2:** Prevalence and characteristics of past year proxy-reported injuries among household members (all ages) of study participants (n = 11186)

	Children (<18 years) n = 6494	Adults (>18 years) n = 4692	Overall
Total number of past year injuries and rate (per 1000)*	142 (22.0)	94 (19.7)	236 (21)

Gender			
Male	94 (29)	64 (26)	158 (28)
Female	48 (15)**	30 (13)**	78 (14)**

SES			
Lower tertile	61 (25)	38 (24)	99 (25)
Middle tertile	53 (23)	31 (18)	84 (20)
Higher tertile	28 (17)	25 (18)	53 (17)

***Place of injury (% of all injuries)***			

Home	74 (52.1)	35 (37.2)	109 (46.2)
Outside	68 (47.9)	59 (62.8)**	127 (53.8)

Type of injury(total number and rate per 1000)*			

Traffic			
Outcome (non-weighted % of total)	47 (6.9)	37 (7.7)	84 (7.3)
Recovery	40 (85.1)	31 (83.8)	71 (84.5)
Disability (temporary)	3 (6.4)	5 (13.5)	8 (9.5)
Disability (prolonged)	3 (6.4)	1 (2.7)	4 (4.8)
Death	1 (2.1)	0 (0)	1 (1.2)

Fall			
Outcome (non-weighted % of total)	31 (5.0)	13 (3.0)	44 (4.2)
Recovery	24 (77.4)	8 (61.5)	32 (72.7)
Disability (temporary)	6 (19.4)	4 (30.8)	10 (22.7)
Disability (prolonged)	1 (3.2)	1 (7.7)	2 (4.5)
Death	0 (0)	0 (0)	0 (0)

Poisoning (food)			
Outcome (non-weighted % of total)	50 (8.0)	32 (6.6)	82 (7.4)
Recovery	50 (100)	32 (100)	82 (100)
Disability (temporary)	0 (0)	0 (0)	0 (0)
Disability (prolonged)	0 (0)	0 (0)	0 (0)
Death	0 (0)	0 (0)	0 (0)

Sharp tool, burn, drowning, electric and others			
Outcome (non-weighted % of total)	14 (2.0)	12 (2.3)	26 (2.2)
Recovery	7 (50.0)	8 (66.7)	15 (57.7)
Disability (temporary)	1 (7.1)	1 (8.3)	2 (7.7)
Disability (prolonged)	6 (42.9)	3 (25.0)	9 (34.6)
Death	0 (0)	0	0 (0)

*All prevalence estimates are calculated from the weighted sample as described in the analysis section.

**p ≤ 0.05 according to Ch^2 ^analysis for the relation between injuries and socio-demographic variables (columns)

**Table 3:** Factors related to injuries in respondents (n= 2038) according to logistic regression analyses

		OR (95% CI)*	*p*
All injuries			
Gender	Female	ref	
	Male	1.4 (1.05–1.96)	0.026
Age	18–29 years	ref	
	30–45 years	1.08 (0.7–1.6)	
	46–65 years	0.62 (0.4–0.95)	0.08

Traffic			
Gender	Female	ref	
	Male	1.7 (0.9–3.1)	0.08

* In each logistic regression model, age, gender, and SES score were entered as independent variables (car ownership was added to the model for traffic injuries)
